# Maternal diabetes induces congenital heart defects in mice by altering the expression of genes involved in cardiovascular development

**DOI:** 10.1186/1475-2840-6-34

**Published:** 2007-10-30

**Authors:** Srinivasan Dinesh Kumar, S Thameem Dheen, Samuel Sam Wah Tay

**Affiliations:** 1 Department of Anatomy, Yong Loo Lin School of Medicine, National University of Singapore, Singapore – 117597

## Abstract

**Background:**

Congenital heart defects are frequently observed in infants of diabetic mothers, but the molecular basis of the defects remains obscure. Thus, the present study was performed to gain some insights into the molecular pathogenesis of maternal diabetes-induced congenital heart defects in mice.

**Methods and results:**

We analyzed the morphological changes, the expression pattern of some genes, the proliferation index and apoptosis in developing heart of embryos at E13.5 from streptozotocin-induced diabetic mice. Morphological analysis has shown the persistent truncus arteriosus combined with a ventricular septal defect in embryos of diabetic mice. Several other defects including defective endocardial cushion (EC) and aberrant myofibrillogenesis have also been found. Cardiac neural crest defects in experimental embryos were analyzed and validated by the protein expression of NCAM and PGP 9.5. In addition, the protein expression of Bmp4, Msx1 and Pax3 involved in the development of cardiac neural crest was found to be reduced in the defective hearts. The mRNA expression of *Bmp4*, *Msx1 *and *Pax3 *was significantly down-regulated (*p *< 0.001) in the hearts of experimental embryos. Further, the proliferation index was significantly decreased (*p *< 0.05), whereas the apoptotic cells were significantly increased (*p *< 0.001) in the EC and the ventricular myocardium of the experimental embryos.

**Conclusion:**

It is suggested that the down-regulation of genes involved in development of cardiac neural crest could contribute to the pathogenesis of maternal diabetes-induced congenital heart defects.

## Background

Maternal diabetes mellitus is associated with a five fold increase in risk of cardiovascular malformations in infants of diabetic mothers [1]. These malformations include endocardial cushion (EC) defects, persistent truncus arteriosus (PTA) and ventricular septal defects (VSD) which appear to result from aberrant cardiac neural crest development [1-3]. Several studies have used animal models to examine the diabetes-induced congenital malformations during development of brain, heart and blood vessels [4-6]. However, the molecular mechanisms by which the teratogenicity of maternal diabetes causes congenital heart defects in embryos remain undefined.

During development, cells from the neural crest over the occipital somites (cardiac neural crest cells; CNCC) migrate to the heart to give rise to parasympathetic postganglionic neurons (cardiac ganglia) as well as ectomesenchymal elements contributing to conotruncal septation in the chick embryo [7,8]. Ablation of the CNCC in chick embryos results in PTA accompanied by a decrease (30%) in the parasympathetic postganglionic innervation of the heart [9]. In mammalian embryos, a similar population of CNCC exists and is perturbed in a number of genetic and teratogenic contexts [10]. Maternal diabetes induced congenital heart defects appear to be similar to the neural crest ablation model [11] since it has been shown that high glucose concentration inhibits migration of neural crest-derived cells from the neural tube in rat embryos [12]. The migration and differentiation of the CNCC into cardiac ganglia are influenced by various developmental control genes. Neural cell adhesion molecule (NCAM) plays a critical role during the development of the avian EC [13]. NCAM expressed early in neural crest formation disappears as migration is initiated and it reappears when CNCC are induced to form cardiac ganglia at the end of their migration [14]. Protein gene product 9.5 (PGP 9.5) appears to be one of the earliest neuron specific genes to be expressed in the developing nervous system. The expression pattern of PGP9.5 closely matches that of the late phase expression of the NCAM [15]. Our recent study in embryonic rat hearts hypothesized that a teratogen such as retinoic acid has an effect on the CNCC resulting in disruption of the differentiation of the cardiac ganglia [16]. In the present study, we speculate that expression of genes controlling normal cardiac ganglia development is altered by maternal diabetes, thereby leading to disruption of the differentiation of the cardiac ganglia.

Maternal diabetes has been shown to induce generalized defects in CNCC development in some, presumably genetically predisposed, individuals among the offspring [17]. Mutations in some genes that control the cardiogenesis result in the congenital heart defects [18-22]. These genes, however have not been linked to congenital heart defects in diabetic offspring. Bone morphogenetic protein 4 (*Bmp4*) has been implicated as regulator of CNCC induction, maintenance, migration, differentiation and survival. It is also required in CNCC for development of the outflow tract (OFT) and ventricular myocardium [23]. It has been shown to be involved in many developmental processes including epithelial mesenchymal transformation (EMT), controls proliferation index in the atrioventricular (AV) cushion during heart development [24,25]. Mutation of *Bmp4 *reduces proliferation index in the AV cushion, leading to AV canal defects [18]. Mouse *Msx1*, which shares homology with the Drosophila muscle segment homeobox (msh) gene, is expressed in a number of non-myocardial cell populations, including cells undergoing an EMT in the AV septum and the OFT region. *Msx1 *has been shown to play an integral role in heart septation and valve formation [26]. Mutation in *Msx1/2 *causes profound deficiencies in the development of structures derived from the cranial and cardiac neural crest [19,20]. The cardiac neural crest-derive aortico-pulmonary septum that divides the distal portion of the OFT into ascending aorta and pulmonary trunk [27]. *Pax3*, a paired-box gene, is required for the migration of CNCC to the developing great vessels. It has been reported that *Pax3 *mutant mice, the naturally occurring splotch mutation, suffer from a PTA [22]. Based on the above facts, it is hypothesized that expression pattern of genes that are involved in the development of cardiac neural crest is altered, leading to congenital heart defects in embryos of diabetic pregnancy. Therefore, the present study was carried out by analyzing the expression pattern of those genes and morphological changes to understand the possible mechanisms causing congenital heart defects in the embryos of diabetic pregnancy.

## Methods

### Experimental Animals

The Swiss Albino mice used in the present study were obtained from the Laboratory Animals Centre, Singapore. Diabetes mellitus was induced in 8 week-old female mice by an intraperitoneal injection of streptozotocin (STZ, 75 mg/kg body weight, Sigma, USA) dissolved in citrate buffer (0.01 M, pH 4.5) on three successive days. Blood glucose levels were examined one week after STZ injection using a Glucometer Elite (Bayer, USA). Only mice with non-fasting blood glucose level exceeding 16 mmol/l were used as experimental diabetic mice. In contrast, control mice maintained the normal blood glucose levels (4–6 mmol/l) before and during pregnancy. Timed mating was carried out by placing four female mice with one normal male mouse in a cage overnight. Noon on the day on which a copulation plug was observed was counted as embryonic day 0.5 (E0.5). On E13.5 (about 60 pairs of somites), pregnant mice were anaesthetized with pentobarbital (150 mg/kg body wt, intraperitoneally) and embryos were collected after Caesarean section. A total of 180 embryos (E13.5) were used for this study. Of these, 120 and 60 embryos were obtained from diabetic (n = 15) and control (n = 6) mothers respectively. The developmental stage, E13.5 was chosen for this study, as it corresponds to ~6 weeks of human gestation [28,29], when all 4 chambers of the heart were clearly distinguishable, and the outflow tract was evident [30]. In addition, the heart phenotype was apparent in embryos from diabetic mice. Embryos with malformations from diabetic mice and normal embryos from non-diabetic mice were used as the experimental and control groups, respectively. All procedures involving animals handling were in accordance with the Principles of Laboratory Animal Care (NIH publication no. 85-23, revised 1985) and guidelines of the Institutional Animal Care and Use Committee (IACUC), National University of Singapore.

### Histology and Immunohistochemistry

Embryonic tissues were fixed with 4% paraformaldehyde (PF) in phosphate buffer (PB) at 4°C overnight, dehydrated and embedded in paraffin. Sections (5 μm thick) were stained with hematoxylin and eosin. For cryostat sectioning, tissues were fixed in 4% PF in PB at 4°C overnight and cryoprotected with 20% sucrose. The method for immunohistochemical analysis was adapted from Kumar and Tay [31]. Transverse or sagittal sections (20 μm thick) of the tissues were cut using a cryostat (Leica CM 3050, Leica Microsystems, Nussloch, Germany), washed with phosphate buffered saline (PBS) and blocked in 5% normal serum for 1 h at room temperature (RT). The sections were then incubated overnight with rabbit polyclonal PGP9.5 (1: 400; UltraClone Ltd, UK) or mouse monoclonal NCAM (1:100; Chemicon International, CA, USA), Bmp4 (1:100; Chemicon International, CA, USA), Msx1 (1: 100; Santa Cruz Biotechnology, Inc) and Pax3 supernatant (1:10; Developmental Studies Hybridoma Bank, University of Iowa) in PBS containing 0.1% Triton X-100 (PBS-TX). Sections were washed in PBS and incubated in the anti-rabbit IgG or anti-mouse IgG for 1 h at RT. The sections were subsequently processed using ABC kit (Vector Laboratories, Burlingame, CA, USA) for 1 h at RT, and reaction products were finally visualized using 3, 3-diaminobenzidine tetrahydrochloride (DAB; Sigma, St. Louis, MO) as the substrate. Tissue sections were counterstained by 0.5% methyl green nuclear stain for 10 minutes, dehydrated by immersion in alcohol and then cleared with xylene before mounting in medium (Permount; Fisher Scientific, Pittsburgh, PA). Control sections were incubated as described above but without the primary antibody. Photomicrographs were taken with a light microscope (Olympus BX51, Olympus, Japan).

### Confocal microscopy

The cryocut sections (20 μm thick) were treated with 1% hydrogen peroxide in methanol to inactivate endogenous peroxidase. The sections were then washed with PBS and incubated overnight with the following primary antibodies: mouse Bmp4 (1:100; Chemicon International, CA, USA), Msx supernatant (1:10; Developmental Studies Hybridoma Bank, University of Iowa) and Pax3 supernatant (1:10; Developmental Studies Hybridoma Bank, University of Iowa) in PBS-TX. Sections were washed in PBS and incubated in the anti-mouse IgG conjugated to FITC (1: 200; Sigma) or anti-mouse IgG conjugated to Cy3 (1:200, Sigma) diluted in PBS-TX accordingly for 1 h at RT. Control sections were incubated as described above but without the primary antibodies. Double immunofluorescence labeling was carried out for the localization of cardiac ganglia and CNCC in the embryonic hearts. Cryosections were rinsed in PBS and incubated with rabbit polyclonal PGP9.5 (1: 400; UltraClone Ltd, UK) and mouse monoclonal NCAM (1:100; Chemicon International, CA, USA) overnight at RT. The sections were then washed and incubated in anti-rabbit IgG conjugated to Cy3 (1:200, Chemicon, USA) and anti-mouse IgG conjugated to FITC (1:200, Chemicon, USA) for 1 h at RT. Sections were finally mounted with fluorescent mounting medium (DAKO, USA). The tissue sections were viewed and photo-images were captured in a confocal laser scanning microscope equipped with a digital camera (Olympus FV1000, Japan).

### Quantitative real-time RT-PCR

Total RNA from three samples at E13.5 of control and diabetic embryonic hearts was extracted using RNeasy mini kit (Qiagen, Hilden, Germany). The reaction mixture containing 2 μg of RNA, 2.5 μM oligo (dT) primer, 200 U of molony murine leukemia virus reverse transcriptase (M-MLV, Promega, USA), 2 mM of each dNTPs, 5 U of RNasin in a total volume of 25 μl was incubated for 1 h at 42°C to synthesize cDNAs. For real time RT-PCR, aliquots (5 μl) of cDNA products were amplified in the reaction mixture (20 μl) containing LightCycler FastStart DNA Master SYBR Green I, 0.5 μM of each primer in a LightCycler instrument (Roche Molecular Biochemicals, Germany) as instructed by the manufacturer. The primers used in this study were shown in Table 1. The fold change of mRNA was analyzed by 2^-ΔΔCt ^method [32].

**Table 1:** Primers and thermal profiles for real-time RT-PCR.

Gene	GenBank Accession No.	Primer	Product size (bp)	Annealing temp./time (°C/s)	Extension temp./time (°C/s)
β-actin	NM_007393	5'-tgttaccaactgggacgaca-3' 5'-ggggtgttgaaggtctcaaa-3'	165	55/7	72/20
Bmp4	NM_007554	5'-tgatacctgagaccgggaag-3' 5'-agccggtaaagatccctcat-3'	190	55/8	72/20
Msx1	NM_010835	5'-agctctgctgccctatacca-3' 5'-cttggcctctgcatccttag-3'	205	57/8	72/20
Pax3	NM_008781	5'-ctgcactcaagggactcctc-3' 5'-gtgaaggcgagacgaaaaag-3'	169	57/7	72/20

### Proliferation index by BrdU labeling

Pregnant mice from the control and diabetic groups (n = 3 in each group) were injected intraperitoneally with BrdU (100 mg/kg body weight, Sigma, USA) 2 h before the embryos were collected by Caesarean section. Frozen sections at the level of the heart were cut (10 μm thick), rinsed in PB (pH 7.4), incubated with 2 mol/l HCl at 37°C for 30 min, then blocked in mouse IgG blocking reagent (Vector Laboratories, Burlingame, CA, USA) for 1 h, and incubated with anti-BrdU monoclonal antibody (1:1000, Sigma, USA) overnight at RT. Subsequently, sections were washed and incubated in biotinylated anti-mouse IgG and ABC kit (Vector Laboratoies, Burlingame, CA, USA). Finally, the reaction products were visualized using DAB (Sigma, St. Louis, MO) as the substrate. The tissue sections were counter-stained with 1% methyl green and mounted in Permount. Photomicrographs were taken with a light microscope (Olympus BX51). The proliferation index was determined by counting the BrdU-positive cells in relation to total cells in every third sagittal section of the heart under a ×20 objective for each group (control and diabetic) in each independent experiments. The proliferation index is expressed as mean ± SD of the percentage of BrdU-positive cells. Identification of cardiomyocytes was confirmed histologically by their characteristic and distinct morphology. The total number of BrdU-positive cells in the embryonic heart between control and diabetic group was compared statistically using Student's *t *test. Differences were considered statistically significant at *p *< 0.05. The procedure used for proliferating cell count in embryos of diabetic mice was previously described [33].

### Detection of apoptosis by TUNEL assay

Terminal deoxynucleotidyltransferase [TdT]-mediated dUTP nick end labelling (TUNEL) assay was performed with an *in situ *cell death detection Kit (Roche), according to the manufacturer's instruction. Briefly, the cryosections (10 μm thick) were incubated with TUNEL reaction mixture containing the terminal deoxynucleotidyltransferase for 60 min at 37°C. Tissues were rinsed with PBS and incubated with DAB substrate for 10 min. The apoptotic cells were counted and the data are presented as the percentage of TUNEL positive cells relative to the total number of cells. The method for apoptotic cell counts in embryos of diabetic mice as described previously [34].

### Transmission electron microscopy (TEM)

The normal embryos from control mice and the defective embryos from diabetic mice (n = 3 in each group) were used for TEM studies. The method for TEM analysis was adapted from Kumar and Tay [35]. Embryos were fixed in 4% PF and 3% glutaraldehyde in PB at 4°C overnight. Briefly, the embryonic heart was dissected into small pieces of tissue samples under the light microscope. The specimens were sampled from the myocardium of entire ventricular wall from both groups. The tissues were then post-fixed in 1% OsO4 in 0.1 M PB for 1 h, dehydrated in a graded series of ethanol and finally embedded in Araldite. Ultrathin sections obtained were double stained with lead citrate and uranyl acetate and viewed under a Philips CM120 electron microscope. The TEM analysis was performed in the embryonic hearts of experimental mice as described previously [36,37].

### Statistical Analysis

The results were analyzed by a two-tailed Student's *t *test using Microsoft Excel software and expressed as means ± SD. Differences were considered statistically significant at *p *< 0.05.

## Results

**Figure 1:**
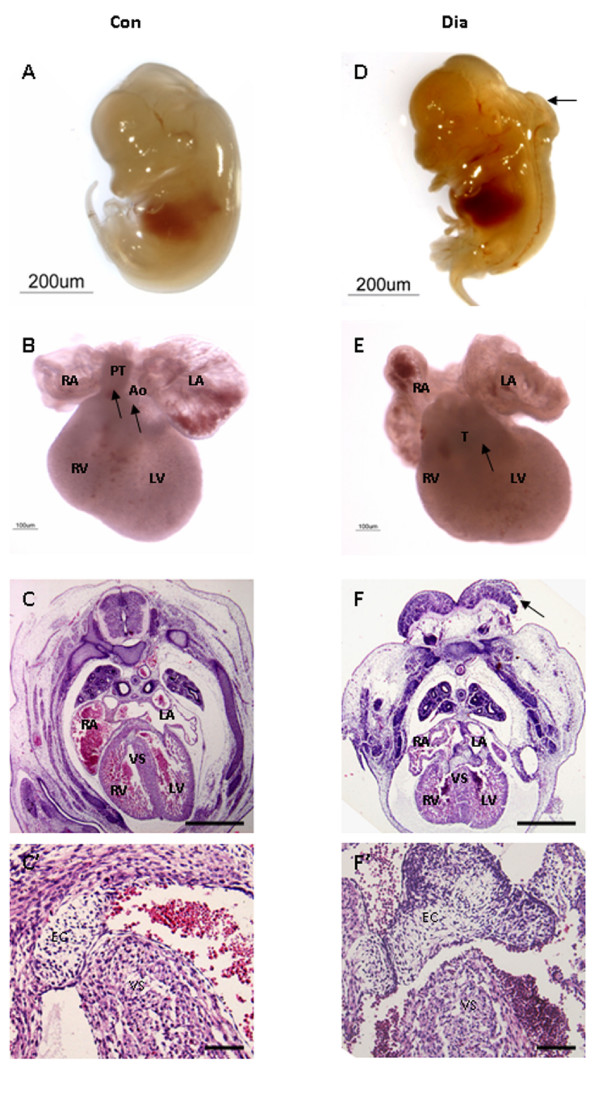
Embryos (E13.5) obtained from control (A-C) and diabetic (D-F) mice. Embryo from control mice shows no gross abnormalities (A). In control embryos, the Ao and PT have separated (*arrows*) and are connected to the LV and RV respectively (connections verified by sectioning, data not shown) (B). In addition, the myocardium and trabeculae are well developed (C). High magnification of the superior aspect of the VS (C'). Embryo from diabetic mice shows neural tube defect (*arrow*) (D). In addition, there is a single outflow vessel (*arrow*), which is wholly committed to the RV (E). Further, the secondary ventricular foramen has failed to closed, histological derangements constituting the myocardium and trabeculae are observed, and the epicardium is disorganized (F). High magnification of the ventricular foramen (F'). *Bar *C, F 500 μm; C', F' 50 μm.

**Figure 2:**
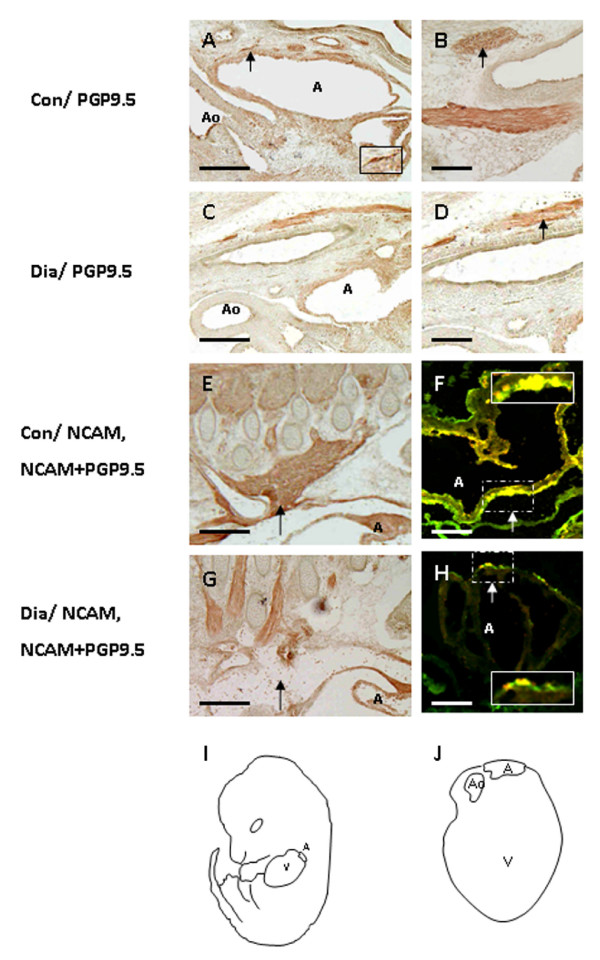
Sagittal sections of the thoracic region of the whole embryos of control (A, B, E, F) and diabetic (C, D, G, H) mice showing the immunoreactivities of PGP9.5 and NCAM. Compared with control (A, B), the number of PGP9.5 positive ganglionic cells appear to be reduced around the great vessels on the posterior atrial wall of experimental embryos (C, D: *arrow*). Inset shows the high magnification of the cardiac ganglionic cell expressing PGP9.5 around the great vessels on the posterior atrial wall (A). The NCAM immunoreactivity appears to be down-regulated in the nerves and ganglia around the atrial epicardium in the diabetic offspring (G, H: *arrow*) as compared with those of controls (E, F: arrow). In addition, the cardiac ganglionic cells expressing PGP9.5 and NCAM immunoreactivity in the atrial epicardium appear to be reduced in embryos of diabetic mice (H: *arrow*) when compared with controls (F: *arrow*). Inset shows high magnification of the cardiac ganglionic cells expressing PGP 9.5 and NCAM immunoreactivities in the atrial epicardium (F, H: *arrow*). Sketch diagrams show the lateral view of the mouse (I) and the sagittal view of the heart (J) indicates in figure 2A-H. *Bar *A, C, E, G 200 μm; B, D, F, H 50 μm.

**Figure 3:**
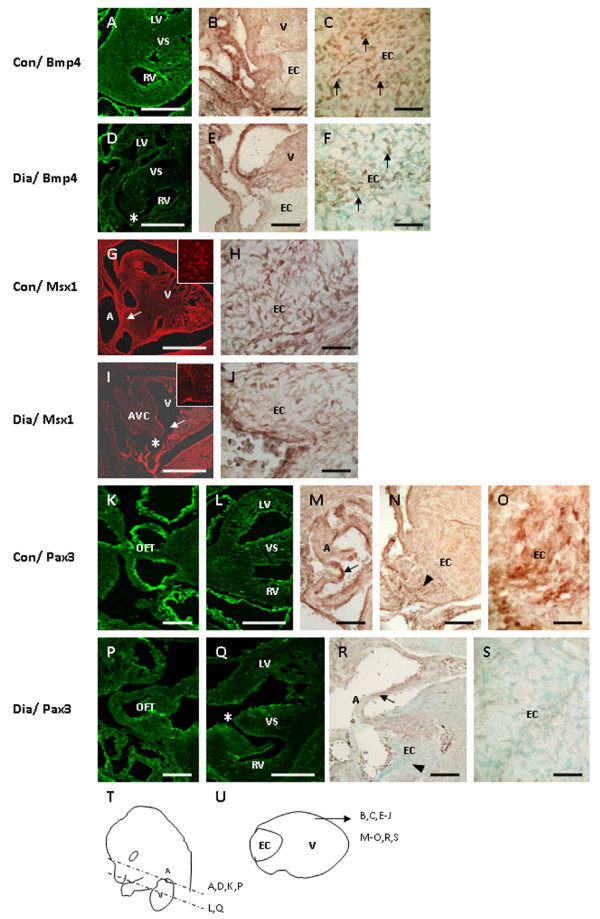
Transverse or sagittal sections show the immunoreactivities for Bmp4 (A-F), Msx1 (G-J) and Pax3 (K-S) in the embryonic heart of control and diabetic mice. Compared with control (A, B, C: *arrows*), the immunoreactivity for Bmp4 appears to be down-regulated in the ventricular myocardium (D), in the OFT (E), and in cardiomyocytes overlying the EC (F: *arrows*) in diabetic offspring. In addition, experimental embryos display VSD (D: *asterisk*). In experimental embryos with AVC defects (I: *asterisk*), the Msx1 expression in endocardial cells in the AVC (I: *arrow*) and in the EC mesenchyme cells (J) appears to be reduced when compared to controls (G, H). Inset shows the area containing the Msx1 positive cells (G, I). In experimental embryos with VSD (Q: *asterisk*), the number of Pax3 positive cells appears to be reduced around the OFT (P), in the atrial wall (r: *arrow*) and in the EC (S: *arrowhead*) as compared with those of controls (K, L, M: *arrow*, N: *arrowhead*, O). Sketch diagrams show the lateral view of mouse (T: *dotted lines indicate the orientation of cut sections in figure 3A, D, K, L, P, Q*) and the sagittal view of the heart (U: *indicate the orientation of cut sections in figure 3B, C, E-J, M-O, R, S*). *Bar *B, C, E, F, H, J, K, M-P, R, S 50 μm; A, D, G, I, L, Q 200 μm

Female Swiss Albino mice were rendered diabetic by STZ at 4 weeks before the beginning of pregnancy. The diabetic female mice were mated with normal male mice and examined daily for the presence of copulation plugs. Only those mice with non-fasting blood glucose level exceeding 16 mmol/l were used as experimental mice. Control animals maintained the normal blood glucose levels (4–6 mmol/l) before and during pregnancy. A total of 180 embryos (E13.5) were used for this study. Of these, 120 and 60 embryos were obtained from diabetic (n = 15) and control (n = 6) mothers respectively (Table 2). The number of embryos per litter was significantly reduced (*p *< 0.001) in diabetic mothers (8 ± 1.2) when compared to controls (10 ± 0.6; Table 2). In addition, the frequency of empty deciduas was found to be 15% in diabetic pregnancies (Table 2). About 10% of embryos from diabetic mice showed a range of cardiac malformations (Table 3). Most of them (7.5%) had a PTA (Fig. 1E) combined with VSD (Fig. 1F, 3D, 3Q). About 2.5% of the embryos showed EC defects (defective AV septum with defective valve development; Fig. 3I) (Table 4). Embryos from diabetic mice also displayed the extra-cardiac malformation (12.5%), such as neural tube defects, including exencephaly, anaencephaly and spina bifida (Table 3). Although embryos of diabetic pregnancies appeared smaller than embryos from non-diabetic pregnancies (as determined by crown-rump length), they were not developmentally delayed (as determined by somite number). The myocardium and trabeculae appear to be disarranged and the epicardium was disorganized in embryos of diabetic pregnancy (Fig. 1F) when compared to controls (Fig 1C). The development of cardiac innervation and ganglionic cells were examined by localization of immunoreactivities of PGP 9.5 and NCAM in embryos from diabetic and control mice. Experimental embryos showed a decrease in the number of cardiac ganglionic cells expressing PGP 9.5 around the great vessels on the posterior atrial wall (Fig. 2C, D) when compared with controls (Fig. 2A, B). The NCAM immunoreactivity appeared to be down-regulated in the nerves and ganglia around the atrial epicardium in embryos of diabetic mice (Fig. 2G, H) when compared with those of the controls (Fig. 2E, F). In addition, the number of cardiac ganglionic cells expressing PGP 9.5 and NCAM immunoreactivities in the atrial epicardium appeared to be reduced in embryos from diabetic mice (Fig. 2H) when compared to controls (Fig. 2F).

**Table 2:** Number (mean ± SD) of embryos and empty deciduas obtained from the control and diabetic mice.

	Control	Diabetes
No. of animals studied	6	15
Total No. of embryos studied	60	120
No. of embryos per litter	10 ± 0.6	8 ± 1.2*
No. of empty deciduas per pregnancy	0	1.2 ± 0.8

* *p *< 0.001 (Student's *t *test)

**Table 3:** Frequencies for cardiac and extra-cardiac malformations in the embryos from control and diabetic mice.

	Control (n = 60)	Diabetes (n = 120)
No. of embryos with cardiac malformations	0	12 (10.0%)
No. of embryos with extra-cardiac malformations^a^	0	15 (12.5%)

^a ^Extra-cardiac malformation such as neural tube defects, including exencephaly, anaencephaly and spina bifida.

**Table 4:** Heart phenotypes of embryos obtained from diabetic mice.

Cardiac malformations	Diabetes n/N (%)
Persistent truncus arteriosus with Ventricular septal defect	9/120 (7.5%)
Defective endocardial cushion	3/120 (2.5%)

n, No. of embryos with cardiac malformations; N, Total No. of embryos studied.

**Figure 4:**
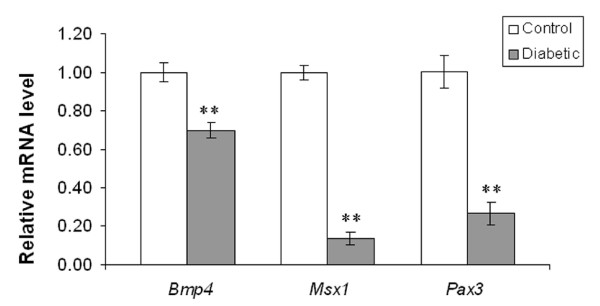
Real-time RT-PCR analysis of Bmp4, Msx1, and Pax3 mRNA expression in the embryonic hearts of control and diabetic mice. Bar graph representing the fold changes of mRNA levels quantified by normalization to the β-actin as an internal control. Mean values ± SD (n = 3). **: *P *< 0.001.

**Figure 5:**
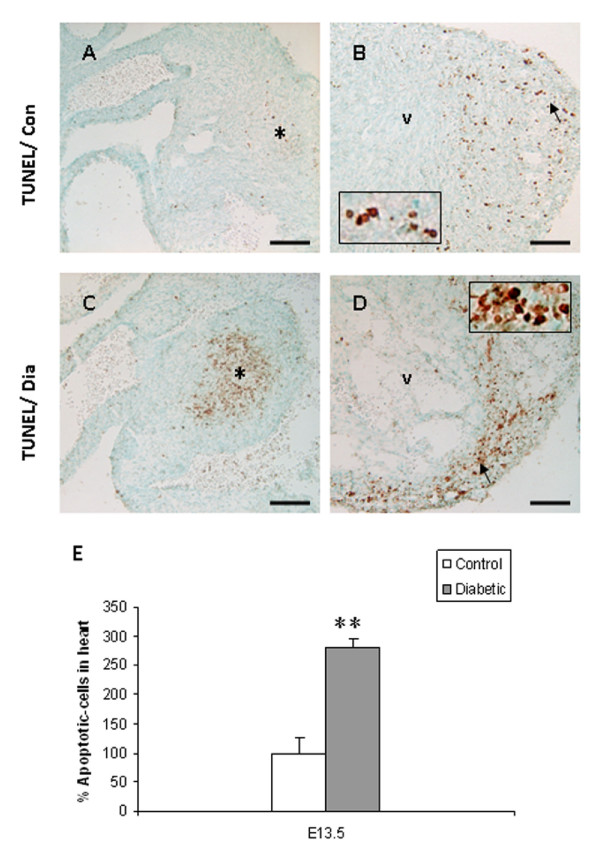
TUNEL assay shows the apoptotic cells in the embryonic heart of control (A, B) and diabetic (C, D) mice. Apoptotic cells appear to be increased in the EC (*asterisk*) and ventricular myocardium (*arrow*) in embryos of diabetic mice (c, d) as compared with those of controls (A, B). Inset shows high magnification of the apoptotic cells in the ventricular myocardium (B, D). Quantitative analysis shows that the apoptotic cells (E) are significantly increased in the developing heart of diabetic mice (filled bar). *Bar *A, B, C, D 50 μm. Mean values ± SD (n = 3). **: *P *< 0.001.

**Figure 6:**
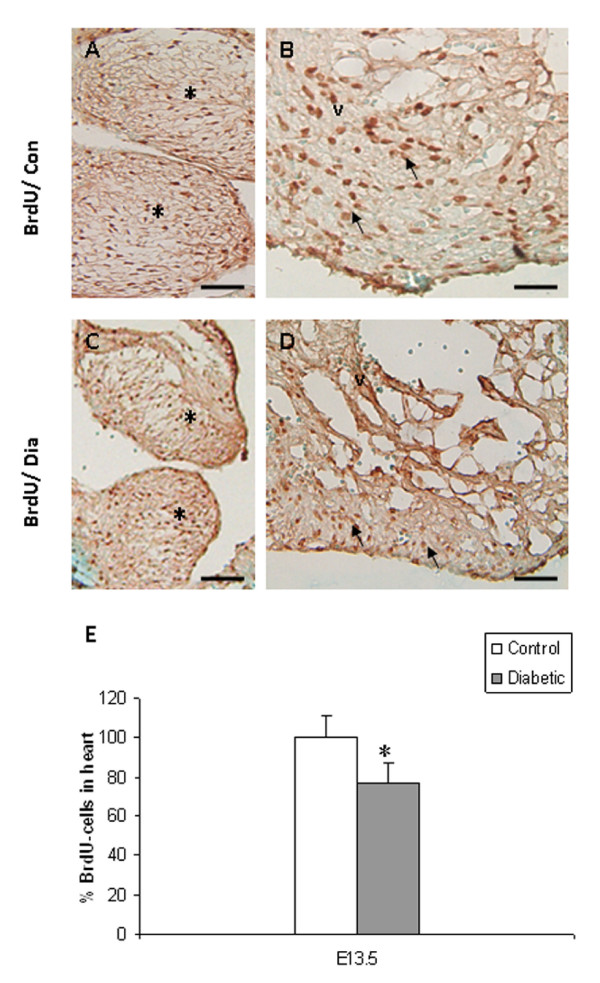
Cell proliferation assay showing the BrdU-labelled cells in the developing heart of embryos from control (A, B) and diabetic (C, D) mice. BrdU-labelled cells appear to be decreased in the EC (*asterisk*) and the ventricular myocardium (*arrow*) in the defective heart of experimental embryos (C, D) when compared to controls (A, B). Quantitative analysis demonstrates that the proliferation index (E) is significantly decreased in the embryonic hearts of diabetic mice (filled bar). *Bar *A, B, C, D 50 μm. Mean values ± SD (n = 3). *: *P *< 0.05.

In embryos of control mice, Bmp4 immunoreactivity was expressed in the muscular layer of the OFT and its derivatives (aorta/pulmonary trunk). It was also detected in cardiomyocytes overlying the EC and in the ventricular septum (Fig. 3a–c), as previously described by Jiao et al. [18]. In contrast, Bmp4 expression was reduced in the OFT and in cardiomyocytes overlying the EC in embryos from diabetic mice (Fig. 3D–F). Real-time RT-PCR analysis revealed a significant decrease in the mRNA expression of *Bmp4 *in embryos of diabetic pregnancy when compared to controls (Fig. 4; *p *< 0.001). Consistent with the histological analysis (Fig. 1F), embryos from experimental mice displayed VSD (Fig. 3D). In normal embryos, Msx1 was strongly expressed in endocardial cells in the AV canal and in the EC mesenchymal cells (Fig. 3G, H). In embryos with AV canal defects from diabetic mice, Msx1 expression was absent in endocardial cells of the AV canal and the number of Msx1 positive mesenchyme cells in the EC was reduced (Fig. 3I, J). Furthermore, the mRNA expression level of *Msx1 *was significantly decreased in embryos of diabetic mice when compared to controls (Fig. 4; *p *< 0.001). In control embryos, Pax3 positive cells were localized around the atrial epicardium, OFT, and in the EC (Fig. 3K–O). In contrast, in embryos with VSD, the number of Pax3 positive cells was notably reduced in the atrial epicardium, OFT and in the EC (Fig. 3P–S). In addition, the mRNA expression level of *Pax3 *was significantly decreased in embryos of diabetic mice when compared to controls (Fig. 4; *p *< 0.001). TUNEL assay showed that the percentage of apoptotic cells in EC and in the ventricular myocardium was significantly increased in the embryos of diabetic mice when compared to controls (Fig. 5A–E; *p *< 0.001). In addition, the total number of BrdU-positive cells in the EC and the ventricular myocardium was significantly decreased in embryos of diabetic mice when compared to controls (Fig. 6A–E; *p *< 0.05).

**Figure 7:**
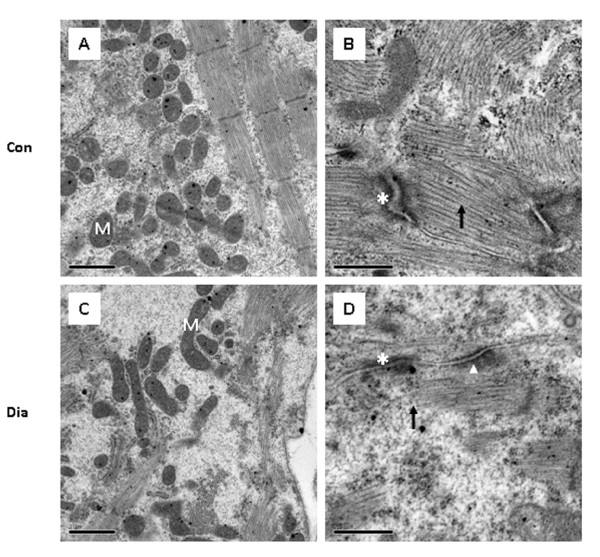
Ultrastructural features of cardiomyocytes in embryos of control (A, B) and diabetic mice (C, D). Some mitochondria appear to be swollen (C) and the number of myofilaments (D: *arrow*) decreased in the ventricular wall of embryos from diabetic mice when compared to controls (A, B). Adherence junctions are less dense (*asterisk*), but normal desmosomes (*arrowhead*), and detachments of myofilaments (D) are found in the ventricular wall of defective heart in embryos of diabetic mice. *Bar *A, C 1 μm; B, D 0.5 μm.

The ultrastructure of cardiomyocytes in the embryos of control mice and the embryos of diabetic mice was also examined (Fig. 7). The cardiomyocytes of the ventricular wall from the malformed heart showed some ultrastructural changes which include swollen mitochondria (Fig. 7C) and decreased number of myofilaments (Fig. 7D) when compared to controls (Fig. 7A, B). In addition, the adherence junctions were poorly developed, with detachments of myofilaments. The desmosomes had developed normally and were indistinguishable from those of the normal controls (Fig. 7D).

## Discussion

Cardiac anomalies are one of the most common malformations in offspring of diabetic mothers [38,39]. This study attempts to reveal the molecular and morphological changes contributing to congenital heart defects in embryos of diabetic pregnancy. The reduction in number of embryos observed in diabetic pregnancies could be due to either reduced fecundity or early embryonic lethality resulting from a higher malformation rate in the embryos of diabetic mothers compared with those of non-diabetic mothers [40]. Recently, an infant born to a type 1 diabetic mother has been shown to exhibit axial mesodermal dysplasia spectrum (AMDS) with AV septal defects. However, molecular mechanisms remain to be undefined [41]. The morphological phenotype observed in maternal diabetes in the present study appear similar to the features of cardiac and extra-cardiac malformations seen in the offspring of diabetic mothers [6,10,33]. The wide variety of cardiac defects observed in the present study suggest a complex pathogenesis, although several of these abnormalities may result from perturbed endothelial-to-mesenchymal transformation or abnormal development and/or migration of CNCC [42].

Maternal diabetes has been shown to induce aberrant CNCC development in diabetic offspring [17]. A recent study also reported that the exposure of CNCC to elevated glucose leads to congenital heart defects [43]. CNCC play multiple roles during heart development and are particularly essential for OFT development. They are also required for regulation of myocardial cell proliferation, as well as differentiation and function of the myocardium [44]. In the present study, maternal diabetes appeared to impair the development of cardiac neural crest-derived cardiac ganglia as revealed by the expression of PGP 9.5 and NCAM. Similar changes have been reported in various neural crest derived tissues in high glucose environment *in vitro *and *in vivo *[45,46]. It seems that the impaired development of cardiac ganglia is due to enhanced maternal diabetes-induced apoptosis and altered expression of genes involved in the differentiation of cardiac ganglia.

Transforming growth factor-β (TGF-β) superfamily has been implicated in promoting NCC induction, maintenance, migration and differentiation in several model organisms [47,48]. *Bmp4 *belonging to the TGFβ superfamily, is a key myocardial signaling molecule which activates EMT during cardiogenesis [49]. *Bmp4 *is also essential for OFT development and regulates a crucial proliferation signal for the ventricular myocardium [23]. Down-regulation of *Bmp4 *expression in cardiomyocytes overlying the EC appears to have contributed to decreased mitotic index in the EC, leading to cardiac defects in the diabetic offspring, since mutation of *Bmp4 *has been shown to reduce proliferation index in AV cushion resulting in AV canal defects in mice [18]. Further, *Bmp4 *expression has been shown to be down-regulated by the *Msx1 *[50], a homeobox gene expressed in AV canal endocardial cells during EMT [51]. The timing of expression and embryonic distribution of *Msx1 *parallels the timing and distribution of *Bmp4 *[45]. A recent study has shown that *Msx1/2 *mutant embryos exhibit defects in the cranial and cardiac neural crest derivatives, including hypoplastic and mispatterned cranial ganglia, dysmorphogenesis of pharyngeal arch derivatives and abnormal organization of conotruncal structures in the developing heart [19]. In this connection, the down-regulation of *Msx1 *expression and the reduced number of Msx1-positive cells in the AV canal and the EC in the developing heart of embryos from diabetic mice observed in the present study may be associated with AV canal and OFT defects. Pax3, which serves as a marker of CNCC, is also essential for the formation of the heart and the OFT in the mouse embryo [21]. Mutation of *Pax3 *in *Splotch *mice perturbs the neural crest affecting development of various structures including the outflow septum of the heart [21,22,52]. The OFT anomaly in *Splotch *mice results from a deficiency in the migration of CNCC [21,52]. In the present study, the mRNA expression of *Pax3 *was significantly down-regulated and the number of Pax3 positive cells were notably reduced in the OFT as well as in the EC in embryos of diabetic mice. This explains the impaired development of CNCC derived tissues (i.e., cardiac ganglia) observed in diabetic offspring. Moreover, the increased number of apoptotic cells in the OFT endocardial cushions in embryos from diabetic pregnancy could be due to the reduced expression of *Pax3 *as down-regulation of *Pax3 *has been shown to cause apoptosis in the developing neural tube of embryos from diabetic mothers [40].

The present report provides the first evidence that ultrastructural changes in cardiomyocytes of the ventricular wall may be associated with the cardiac defects observed in embryos of diabetic pregnancy. The ultrastructural changes in cardiomyocytes of embryos from diabetic mice were characterized by the presence of swollen mitochondria and disorganized myofilaments with poorly developed adherence junctions and have been consistent with previous observation in mutant mice [36]. In addition, the abnormal adherence junctions with detachment of myofilaments are as reported previously in some transgenic mice [37]. This suggests that aberrant myofibrillogenesis could lead to histological derangements in the developing heart of diabetic pregnancy [36].

## Conclusion

The present study demonstrates that maternal diabetes alters the expression of some genes that are involved in the development of the cardiac neural crest, leading to congenital heart defects.

## Abbreviations

A: atrium; Ao: aorta; AV: atrioventricular; AVC: AV canal; AMDS: axial mesodermal dysplasia spectrum; CNCC: cardiac neural crest cells; E: embryonic day; EC: endocardial cushion; EMT: epithelial mesenchymal transformation; LA: left atrium; LV: left ventricle; M: mitochondria; OFT: outflow tract; PT: pulmonary trunk; PTA: persistent truncus arteriosus; RA: right atrium; RV: right ventricle; STZ: streptozotocin; T: single outflow vessel; V: ventricle; VS: ventricular septum; VSD: ventricular septal defects.

## Competing interests

The author(s) declare that they have no competing interests.

## Authors' contributions

The study was designed by SDK, STD and SSWT. SDK carried out all the experiment, interpreted the data and drafted the manuscript. STD and SSWT participated in the preparation of the manuscript. SSTW is the Principal Investigator for the Biomedical Research Council (BMRC) research grant. All authors read and approved the final manuscript.
